# Alterations of Glucose-Dependent Insulinotropic Polypeptide and Expression of Genes Involved in Mammary Gland and Adipose Tissue Lipid Metabolism during Pregnancy and Lactation

**DOI:** 10.1371/journal.pone.0078560

**Published:** 2013-11-13

**Authors:** R. Charlotte Moffett, Nigel Irwin, Jacqueline M. E. Francis, Peter R. Flatt

**Affiliations:** School of Biomedical Sciences, University of Ulster, Coleraine, County Londonderry, United Kingdom; University of Lancaster, United Kingdom

## Abstract

Gastric inhibitory polypeptide (GIP) is a gut derived peptide with multiple emerging physiological actions. Effects of pregnancy and lactation on GIP secretion and related gene expression were studied in Wistar rats. Pregnancy moderately increased feeding (p<0.05), whilst lactation substantially increased food intake (p<0.01 to p<0.001). Circulating GIP was unchanged during pregnancy, but non-fasting plasma glucose was significantly (p<0.01) decreased and insulin increased (p<0.05). Lactation was associated with elevated circulating GIP concentrations (p<0.001) without change of glucose or insulin. Oral glucose resulted in a significantly (p<0.001) decreased glycaemic excursion despite similar glucose-induced GIP and insulin concentrations in lactating rats. Pregnant rats had a similar glycaemic excursion but exhibited significantly lowered (p<0.05) GIP accompanied by elevated (p<0.001) insulin levels. Pregnant rats exhibited increased (p<0.001) islet numbers and individual islet areas were enlarged (p<0.05). There were no significant differences in islet alpha-cell areas, but all groups of rats displayed co-expression of glucagon and GIP in alpha-cells. Lactating rats exhibited significantly (p<0.01) increased intestinal weight, whereas intestinal GIP stores were significantly (p<0.01) elevated only in pregnant rats. Gene expression studies in lactating rats revealed prominent (p<0.01 to p<0.001) increases in mammary gland expression of genes involved in energy turnover, including GIP-R. GIP was present in intestines and plasma of 17 day old foetal rats, with substantially raised circulating concentrations in neonates throughout the period of lactation/suckling. These data indicate that changes in the secretion and action of GIP play an important role in metabolic adaptations during pregnancy and especially lactation.

## Introduction

Gastric inhibitory polypeptide (GIP) is a key incretin hormone that regulates post-prandial glucose homeostasis [1]. Besides well characterised nutrient-dependent insulinotropic effects, GIP has actions outside of the pancreas [2], as evidenced through widespread tissue GIP receptor expression [3]. Thus, GIP has important regulatory effects on bone turnover, lipid metabolism and energy regulation [4]–[7]. Once released into the blood stream GIP exerts overall anabolic effects, favouring energy and nutrient deposition [8], [9]. Importantly, the secretion of GIP from intestinal K-cells is tightly controlled by absorption of the digestion products of carbohydrate, protein and particularly fat from the small intestine [10].

Pregnancy and the transition to lactation are physiological states where energy balance is subjected to major metabolic demands [11]. Thus, nutritional requirements are greatly increased to support the development of the foetus and the subsequent nourishment of the new-born by milk production [12]. It is reasoned that the accompanying hyperphagia should also increase the function of the intestinal tract and the secretion and subsequent action of gut related peptides [13]. Consistent with this view, there is a large proliferation intestinal mass during pregnancy and lactation [14]. Accompanying changes in the biological actions of gut derived hormones, such as GIP, are likely to play a key role in the metabolic adaptations imposed by pregnancy and lactation. Despite this, alterations of intestinal K-cell function and GIP secretion and action are not well documented during pregnancy or lactation.

Glucose homeostasis and insulin sensitivity are modified in pregnancy and lactation [15]. Pregnancy is associated with insulin resistance and increased insulin demand whereas lactation results in improved insulin action [16]. In this context, gut derived peptides such as GIP, have well known effects on insulin secretion and sensitivity, as well as body weight control and adipose tissue metabolism [1], [9], [17], [18]. Thus, GIP could also be partly responsible for the altered glucose homeostasis, insulin sensitivity and changes of energy metabolism observed during pregnancy and lactation [15]. Moreover, during pregnancy pancreatic beta-cells undergo major up-regulatory structural and functional changes in response to the increased demand for insulin, including expansion of beta-cell mass [19]. Given that GIP is an important growth and anti-apoptotic factor for beta-cells [20], [21], it may also play a role in the compensatory islet response to pregnancy.

Therefore, the present study has investigated changes in GIP synthesis and secretion in the context of metabolic adaptations that occur during pregnancy and lactation. We have monitored circulating GIP concentrations, intestinal tissue GIP stores as well as pancreatic islet morphology and possible co-expression of GIP in glucagon containing alpha-cells in pregnant and lactating Wistar rats. Related effects on glucose homeostasis and insulin secretion were also considered. In addition, we examined the effects of pregnancy and lactation on the expression of genes involved in energy turnover in both abdominal adipose and mammary tissue. Finally, we have monitored circulating and intestinal GIP in offspring during foetal and neonatal development. The results suggest an important role of GIP in metabolic adaptations during pregnancy and lactation.

## Materials and Methods

### Animals

Female, virgin, albino Wistar rats (15 weeks old) were obtained from Harlan Ltd. UK. Animals were housed singly in an air-conditioned room at 22±2°C with a 12 h light:12 h dark cycle (08:00–20:00 h). Drinking water and a standard rodent maintenance diet (10% fat, 30% protein and 60% carbohydrate, Trouw Nutrition, Cheshire, UK) were provided ad libitum. All animal experiments were carried out in accordance with the UK Animals (Scientific Procedures) Act 1986 and approved by the University of Ulster Animal Ethics Review Committee. All necessary steps were taken to ameliorate any potential animal suffering and animals were sacrificed by lethal inhalation of CO_2_ followed by cervical dislocation.

### Experimental Protocols for *in vivo* studies

Groups of female rats were time-mated and caged individually. Pregnancy proceeded without intervention until parturition, at which point litter sizes were standardised to n = 10. Food intake, body weight, non-fasting plasma glucose, insulin and GIP concentrations were monitored at 4–7 day intervals. On day 21 of both pregnancy and lactation, an oral glucose tolerance (3.2 g/kg body weight (51.2 kJ/kg)) and oral fat (1.38 g corn oil/kg body weight (51.2 dkJ/kg)) challenge were performed in two groups of rats following an 18 h fast. At the end of the study, small intestines were excised, weighed and processed for measurement of GIP following extraction with 5 ml/g of ice-cold acid ethanol (750 ml ethanol, 235 ml water, 15 ml conc HCl). In a separate series, small intestines were similarly processed from foetal rats or neonates on days 10, 14, 17, 19, 20 and 21 of intrauterine life and days 1, 2, 3, 7, 10, 14, 17, 20, 22, 23, 25, 38 and 45 following birth.

### Histology and immunostaining

For morphological analysis, at the end of respective studies, pancreata were excised and fixed in 4% (w/v) paraformaldehyde/PBS and embedded in paraffin. Slides (8 µm) were stained using monoclonal mouse anti-insulin (1∶500; Sigma-Aldrich, Poole, UK) and Alexa Fluor 594 anti-mouse (1∶400; Invitrogen, Paisley, UK) antibodies. For fluorescence microscopy, highly specific polyclonal guinea pig anti-glucagon (PCA2/4; 1∶400 [22]) and polyclonal rabbit anti-GIP (RIC34/111J; 1∶400; courtesy of Professor Linda Morgan, University of Surrey) antibodies were used together with visualization using donkey anti-guinea pig Alexa Fluor 594 and donkey anti-rabbit Alexa Fluor 488 (both from Invitrogen, Paisley, UK), respectively. All analyses of sections were performed using Image J software [23]. Approximately 60–70 random sections were examined from the pancreas of each rat.

### Biochemical analysis

Blood samples (∼0.4 mL) taken from the cut tip of the tail of conscious rats or by decapitation from foetal/neonatal rats at the times indicated in the Figures and were immediately centrifuged using a Beckman microcentrifuge (Beckman Instruments, UK) for 30 s at 13,000 ***g***. The resulting plasma was aliquoted into fresh Eppendorf tubes and stored at −20°C until analysis. Plasma glucose was measured by an automated glucose oxidase procedure using a Beckman Glucose Analyzer II. Plasma insulin was assayed by a modified dextran-coated charcoal radioimmunoassay [24]. Plasma and tissue GIP were measured by radioimmunoassay using rabbit anti-porcine GIP antiserum (RIC34/111J) as originally described by Morgan *et al*. [25]. The antiserum reacts against the C-terminus of GIP, thereby measuring ‘total’ GIP concentrations [26]. A small amount of plasma could be obtained from foetal rats from day 14 of gestation onwards, but only from day 17 onwards was there sufficient for GIP assay.

### Gene expression

At the end of pregnancy and lactation, mammary and abdominal adipose tissue (n = 4) was excised and immediately snap-frozen in liquid nitrogen and stored at −80°C before RNA extraction for gene expression analysis. Briefly, total RNA was isolated and purified using QIAzol lysis reagent (Qiagen, West Sussex, UK) and RNA concentration determined from the absorbance at 260 nm. First-strand cDNA was synthesised using 2 μg of total RNA at 42°C for 50 min in the presence of 0.5 μg oligo dT(12–18) primer, 10 mM dNTP and 200 U Superscript II reverse transcriptase (Invitrogen, Paisley, UK) in a final volume of 20 μl using a GeneStorm GS1 Thermal Cycler (Gene Technologies Ltd, Essex, UK). Genes were amplified using specific primers for β-actin (reference gene), acetyl CoA carboxylase-1 (ACC-1), oestrogen receptor A (ESS-RA), oestrogen receptor B (ESS-RB), fatty-acid transport protein (Fat-P), glucagon receptor (GCG-R), GIP receptor (GIP-R), glucose transporter type 4 (GLUT4), 11β-hydroxysteroid dehydrogenase type 1 (HSD-1), hormone-sensitive lipase (HSL), lipoprotein lipase (LPL) and prolactin receptor (Prl-R). Primer sequences are shown in Table 1. The DNA-denaturing step was carried out at 95°C for 5 min in a Roche LightCycler 1.5 carousel-based thermal cycler (Roche Diagnostics, West Sussex, UK). cDNA amplification then commenced for 40 cycles with 95°C denaturation for 30 s, 58°C annealing for 30 s and 72°C elongation for 30 s with SYBR green fluorescence being read after each cycle and recorded by Roche LightCycler Software (Version 3.5) to construct an amplification curve. Gene expression was calculated from 2^Δ^C_t_ values normalised to Atcb control primer. Age-matched normal female, virgin, albino Wistar rats were used for comparative purposes.

**Table 1:** Primer sequences.

Gene	Forward	Reverse	UPL Number
ESS-RA	CCT GGT CTG TGG GGA TGT	GGA CAG CTG TAC TCG ATG CTC	106
ESS-RB	GCC CTT GCC AAC TCA GAT T	TGG CTC AGC TCA TAG TCC TG	62
Prl-R	CAG TGG CTT TGA AGG GCT AT	TCC AGC AGA TGG GTA TCA AA	31
LPL	GAA ATG ATG TGG CCA GGT TC	TGG ACA TTG TCT AGG GGG TAG T	69
Fat-P	GGA CCA CCG GAC TTC CTA AG	GAA GGC TGC AAT GCG GTA	62
GLUT4	TGC AGT GCC TGA GTC TTC TTT	CCA GTC ACT CGC TGC TGA	120
ACC-1	CAT CAC ATC GGT CCT GTG TC	GCT GCA TGA CTA TCT AGG ATG TTG	20
GIP-R	TGG TAT TTG CTC CCG TGA C	AGC ACA CTC ACG AGG AAA CC	41
HSL	CGA GCA CTG GAG GAG TGT TT	TAT CCG CTC TCC GGT TGA	3
HSD-1	AAA CAG AGC AAT GGC AGC AT	CAG AGG TTG GGT CAT TTT CC	25
GCG-R	CCA GTG CCA CCA CAA CCT A	AGT TCT GTT GCA GAC CAG CTC	74

UPL; Universal Probe Library.

### Statistics

Results are expressed as mean ± S.E.M. Data were compared using ANOVA, followed by a Student-Newman-Keuls *Post hoc* test. Area under the curve (AUC) analyses were calculated using the trapezoidal rule with baseline subtraction. Comparisons with p<0.05 were considered to be statistically significant.

## Results

### Effects of pregnancy and lactation on body weight, food intake and non-fasting glucose, insulin and GIP levels

**Figure 1:**
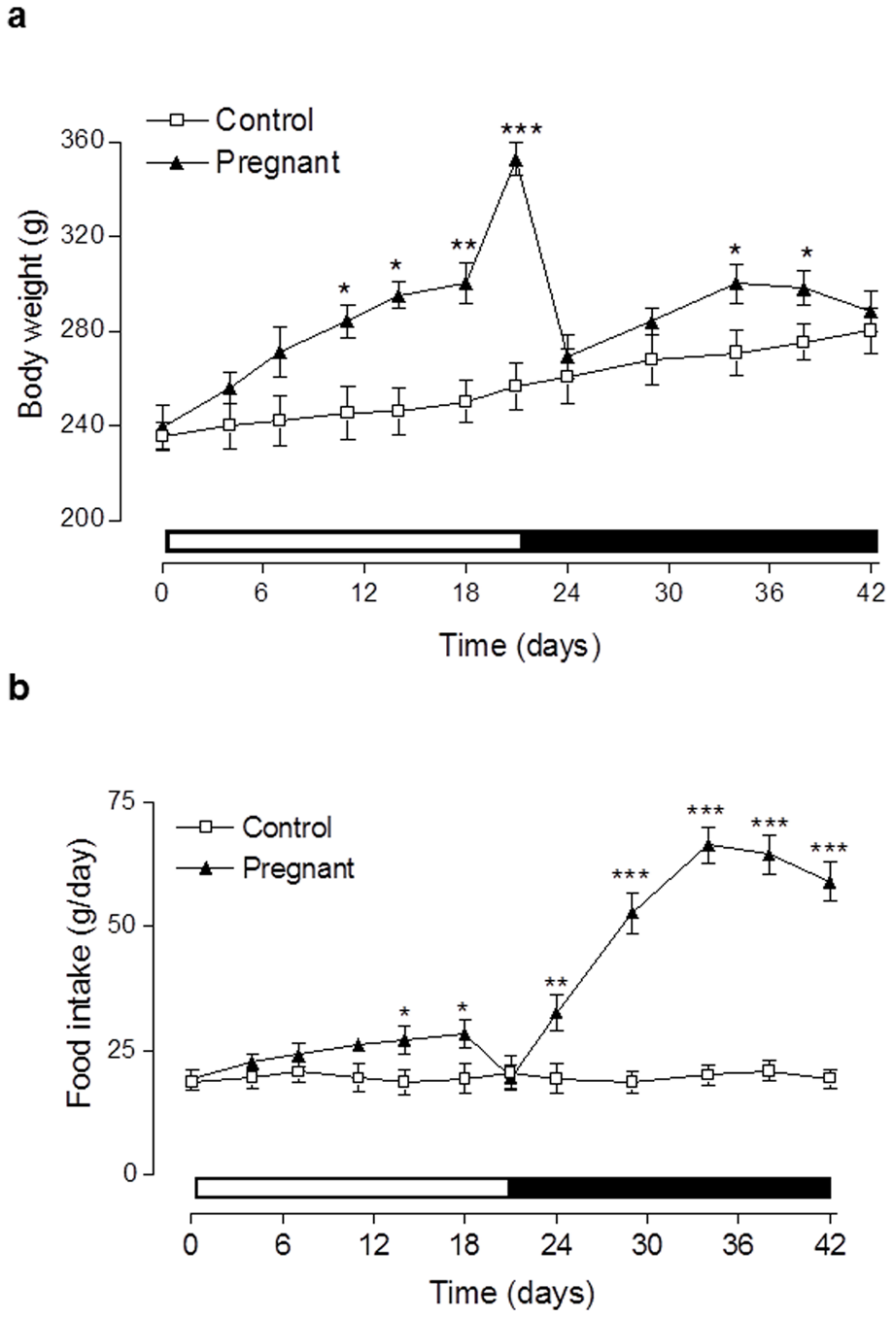
Effects of pregnancy and lactation on (a) body weight and (b) food intake in Wistar rats. Parameters were measured for 21(indicated by open bar) and 21 days subsequent to parturition (indicated by black bar). Values are means ± SEM for 6 rats. * p<0.01, **p<0.01 and *** p<0.001 compared to controls.

**Figure 2:**
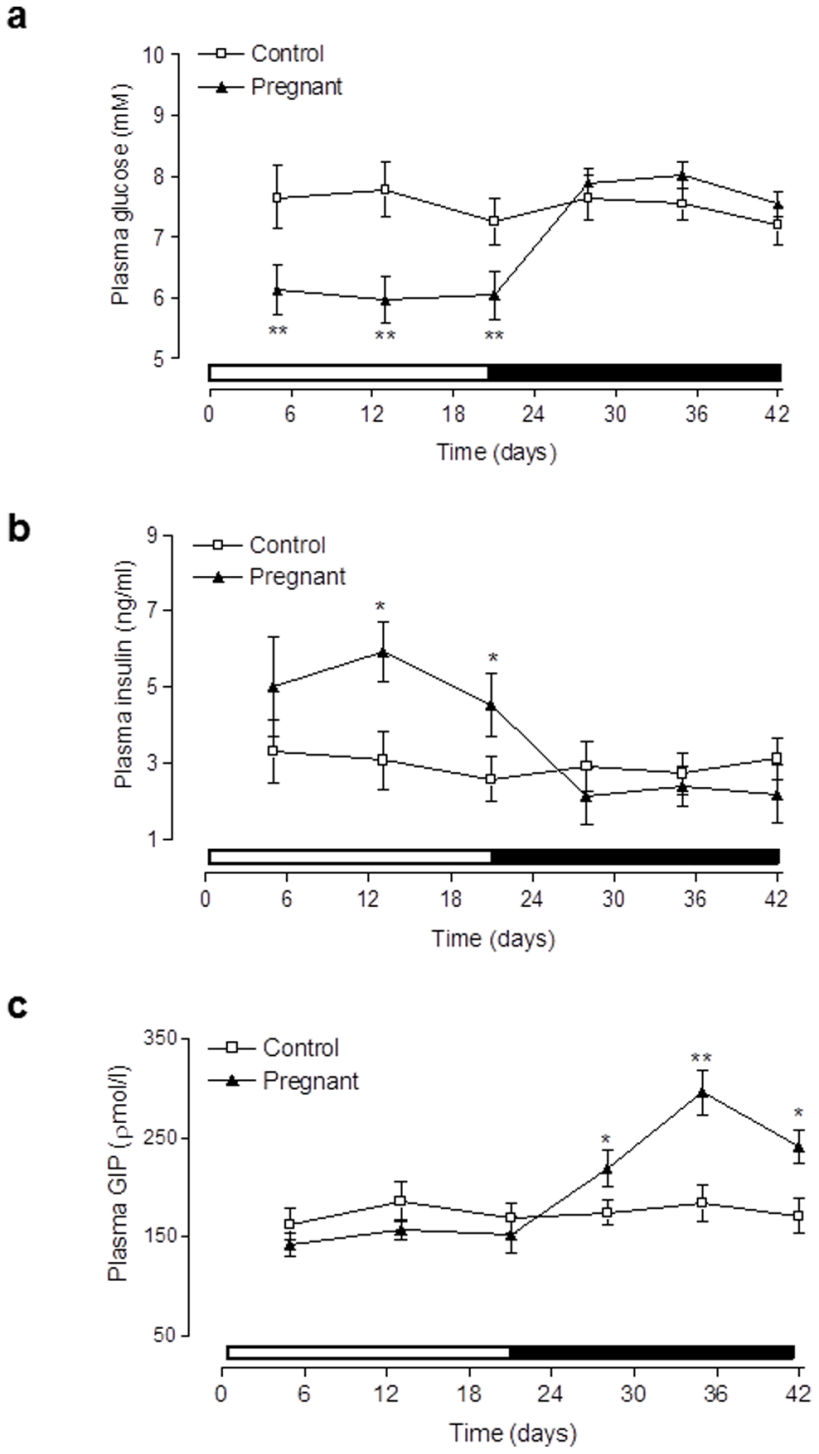
Effects of pregnancy and lactation on non-fasting plasma (a) glucose, (b) insulin and (c) GIP in Wistar rats. Parameters were measured for 21(indicated by open bar) and 21 days subsequent to parturition (indicated by black bar). Values are means ± SEM for 6 rats. * p<0.01 and ** p<0.01 compared to controls.

As shown in Figure 1, body weight of pregnant rats was significantly (p<0.05 to p<0.001) increased from 13 days post coitus until parturition on day 21, when compared to controls (Figure 1A). Body weights of lactating rats then rapidly returned to near control levels 3 days after parturition (day 24). However, on days 12 and 16 of lactation there was a transient increase (p<0.05) in body weight compared to control rats (Figure 1A). This elevation of body weight in lactating rats was associated with dramatic and significant (p<0.01 to p<0.001) increases in food intake compared to control rats (Figure 1B). In addition, pregnant rats also demonstrated significantly (p<0.05) increased food intake on days 14 and 18 post coitus when compared to controls (Figure 1B). Non-fasting plasma glucose levels of pregnant rats were significantly decreased (p<0.01) compared to controls at each time point tested (Figure 2A). In contrast, during the lactation phase, plasma glucose levels were similar to control rats (Figure 2A). In agreement with this, pregnant rats exhibited significant (p<0.05) elevations of non-fasting plasma insulin levels on day 13 and 21, that were returned to control levels following parturition (Figure 2B). Interestingly, pregnancy was associated with remarkably similar, whilst lactation induced significantly elevated (p<0.05 to p<0.01), non-fasting circulating GIP levels compared to control rats (Figure 2C). Calculation of non-fasting glucose: insulin ratio revealed pregnant rats had significantly (p<0.01) decreased values (1.05±0.3) compared to lactating and control rats (2.72±0.4 and 3.09±0.4; respectively), indicative of impaired insulin sensitivity.

### Effects of pregnancy and lactation on oral glucose tolerance and glucose-stimulated plasma insulin and GIP levels

**Figure 3:**
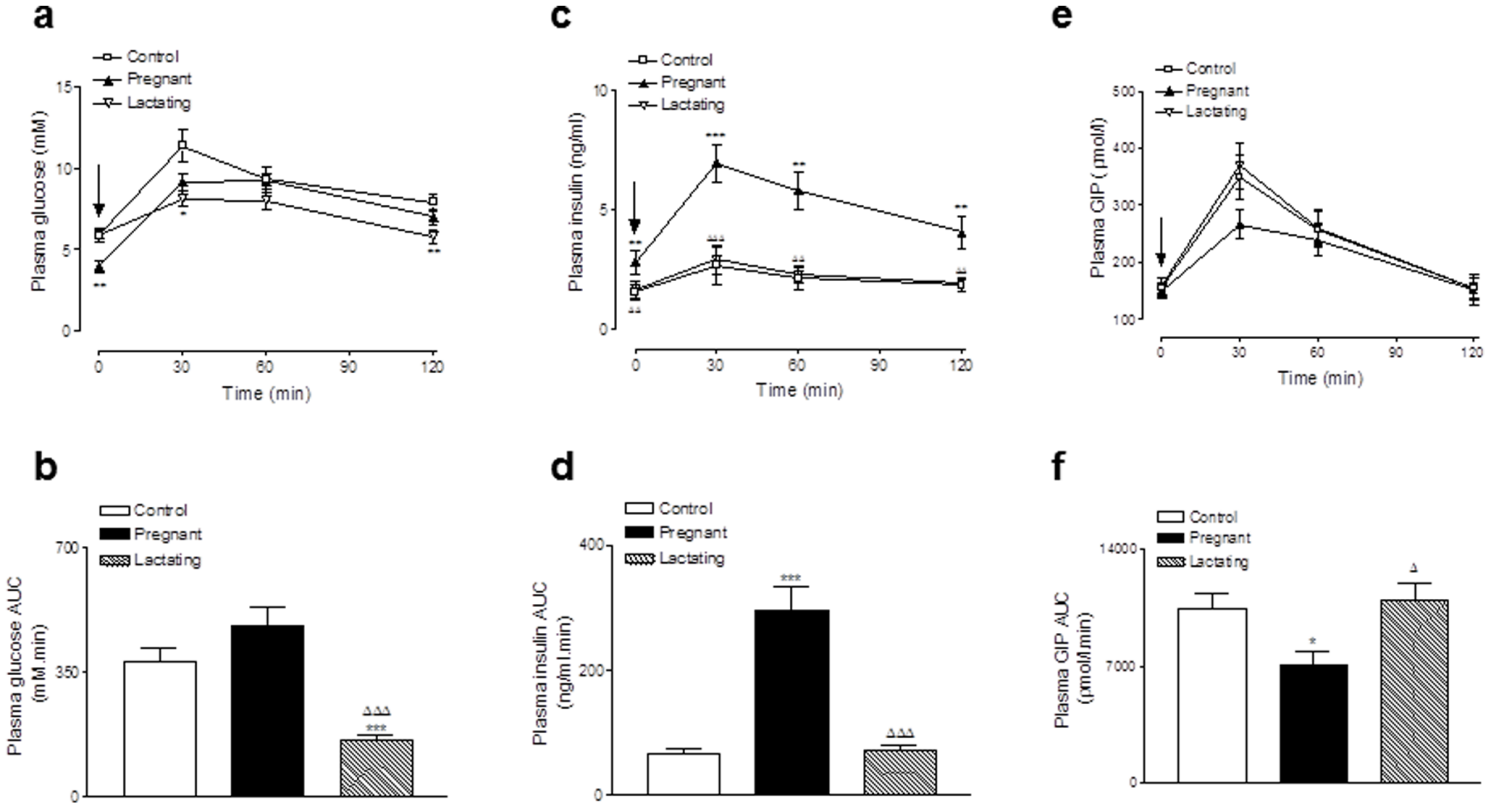
Effects of pregnancy and lactation on (a,b) glycaemic, (c,d) insulin and (e,f) GIP responses to oral glucose in Wistar rats. Tests were conducted on days 21 and 42 in overnight fasted rats. Oral glucose (51.2 kJ/kg body weight) was administered at the time indicated by the arrow. Plasma glucose, insulin and GIP AUC values for 0–120 min are also shown Values are means ± SEM for 6 rats. * p<0.01, ** p<0.01 and *** p<0.001 compared to controls. ^Δ^ p<0.05, ^ΔΔ^ p<0.01 and ^ΔΔΔ^ p<0.001 compared to pregnant rats.

Lactating rats had significantly (p<0.05) decreased plasma glucose levels at 30 and 120 minutes post glucose administration when compared to control rats. This was corroborated in the 0–120 min AUC values with lactating rats having a significantly (p<0.001) reduced overall glycaemic excursion compared to control rats (Figure 3). However, individual and overall AUC glucose-stimulated plasma insulin levels were not significantly different when compared to control rats (Figure 3). Pregnant rats exhibited significantly (p<0.01) decreased plasma glucose levels and elevated plasma insulin levels prior to glucose administration (Figure 3). However, pregnant rats displayed a similar overall 120 min glycaemic excursion compared to controls but markedly (p<0.001) elevated overall glucose-induced insulin concentrations (Figure 3). Individual GIP concentrations were not significantly different between groups, however overall glucose-induced AUC plasma GIP levels were significantly (p<0.05) decreased in pregnant, but not lactating, rats when compared to controls (Figure 3).

### Effects of pregnancy and lactation on plasma glucose, insulin and GIP levels after oral fat

**Figure 4:**
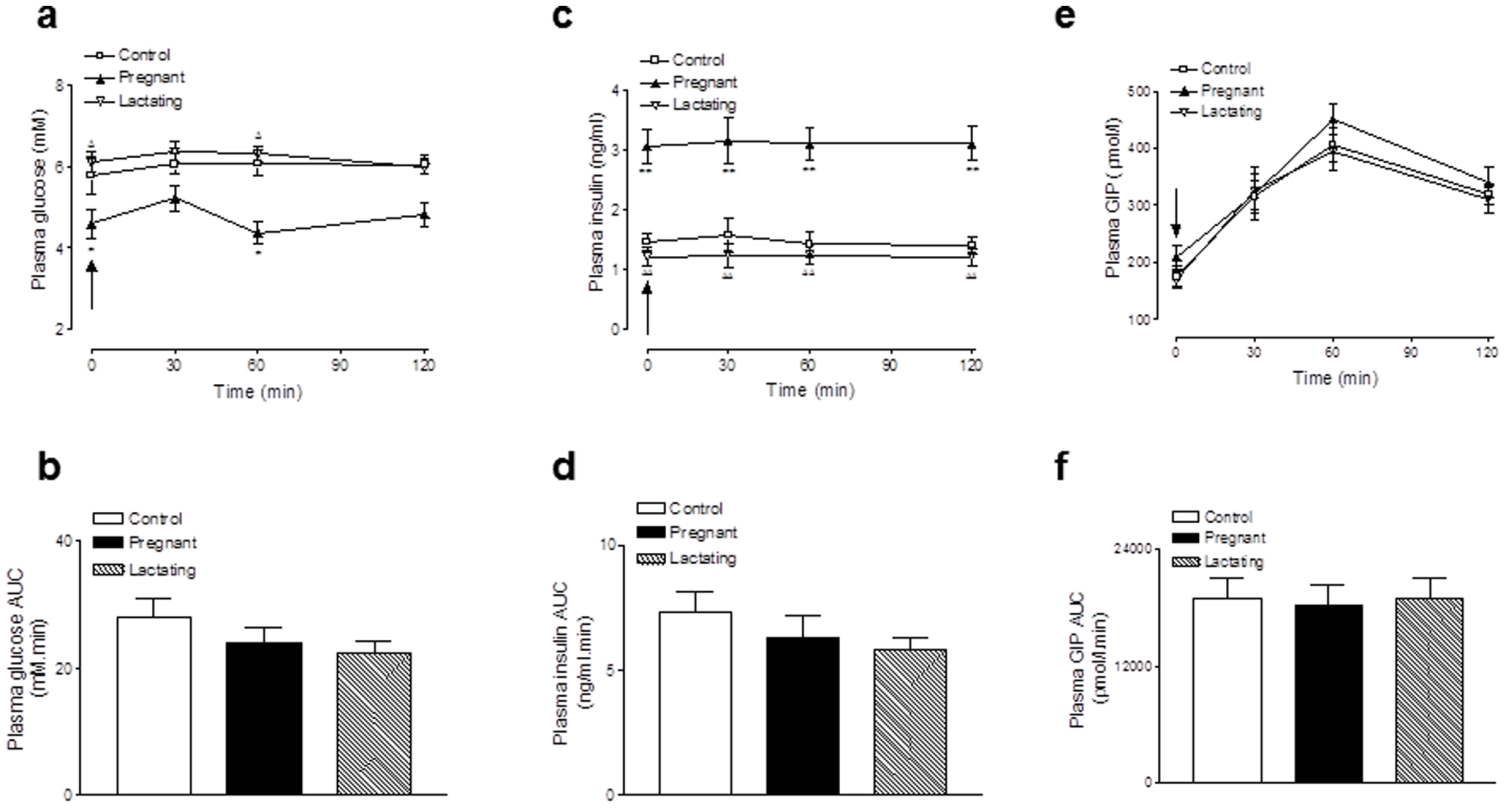
Effects of pregnancy and lactation on (a,b) glycaemic, (c,d) insulin and (e,f) GIP responses to oral fat. Tests were conducted on days 21 and 42 in overnight fasted rats. Oral fat (51.2 kJ/kg body weight) was administered at the time indicated by the arrow. Plasma glucose, insulin and GIP AUC values for 0–120 min are also shown. Values are means ± SEM for 6 rats. * p<0.01 and ** p<0.01 compared to controls. ^Δ^ p<0.05 and ^ΔΔ^ p<0.01 compared to pregnant rats.

Figure 4 depicts the effects of acute oral fat administration in pregnant, lactating and control rats. Administration of oral fat resulted in prominent and similar increases in GIP concentrations in all groups (Figure 4). In addition, there was no significant effect on overall plasma glucose or insulin levels in pregnant or lactating rats when compared to controls (Figure 4). Plasma glucose levels were significantly lower (p<0.05) and insulin levels higher (p<0.01) pre-dosing in pregnant rats compared to controls, and remained as such during the 120 min observation period (Figure 4).

### Effects of pregnancy and lactation on pancreatic morphology

**Figure 5:**
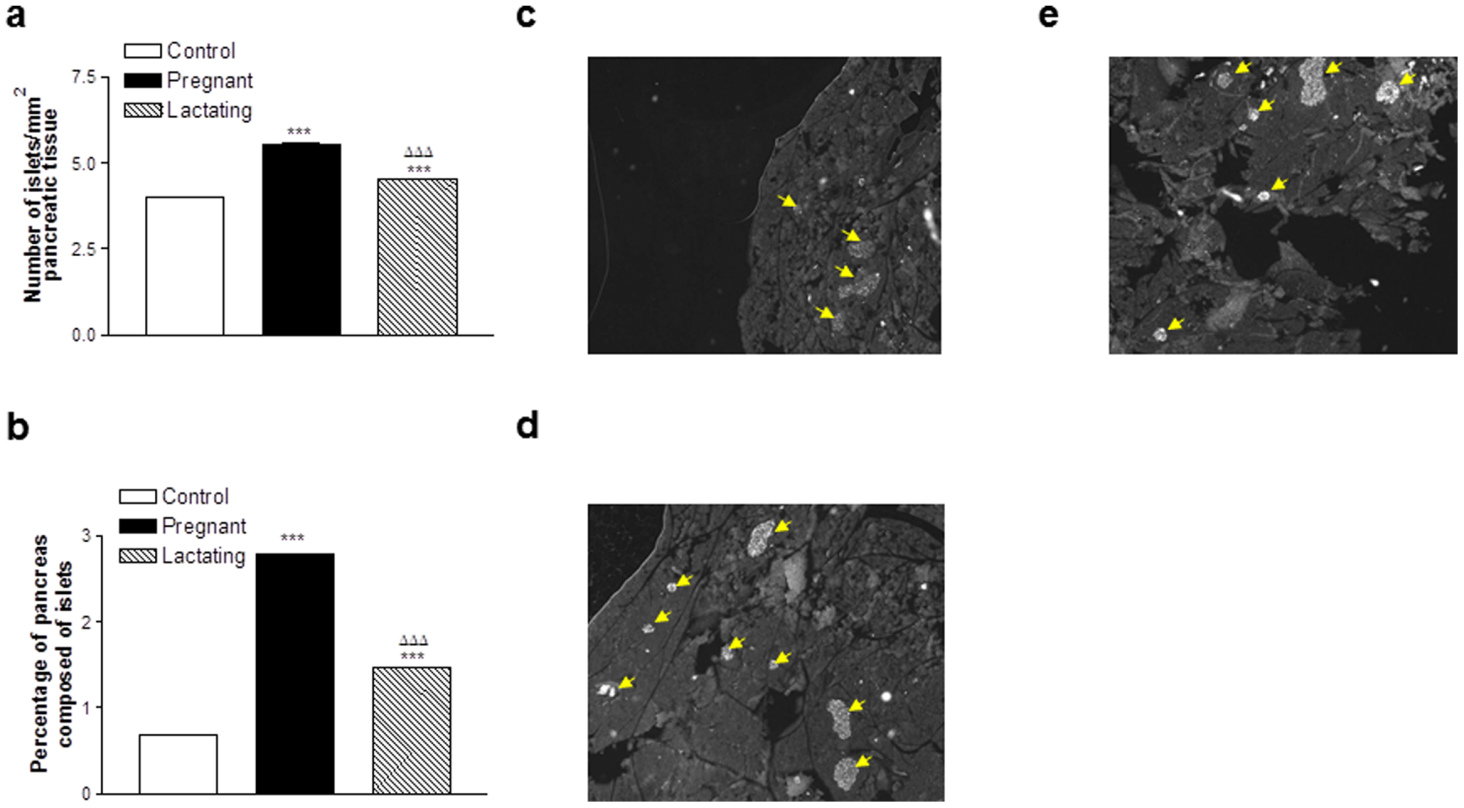
Effects of pregnancy and lactation on pancreatic islet (a) number, (b) density and (c–e) pancreatic morphology in Wistar rats. Parameters were measured on days 21 and 42. (**c–e**) Images with immunofluorescent insulin staining are shown for (**c**) control, (**d**) pregnant and (**e**) lactating rats, with islets indicated by the arrows. Values are means ± SEM for 3–4 rat pancreata. *** p<0.001 compared to controls. ^ΔΔΔ^ p<0.001 compared to pregnant rats.

**Figure 6:**
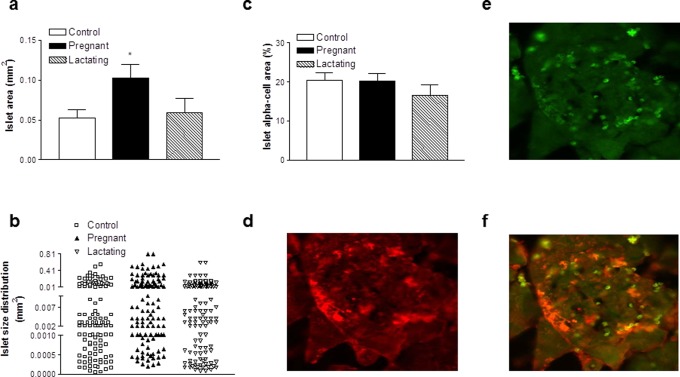
Effects of pregnancy and lactation on pancreatic islet (a) area, (b) size distribution, (c) alpha-cell content and (d–f) specific immunohistochemical staining for (d) GIP, (e) glucagon and (f) GIP and glucagon of pancreatic islets in Wistar rats. (**a–c**) Parameters were measured on days 21 and 42. (**d–f**) Images are shown for lactating rats. Values are means ± SEM for 3–4 rat pancreata. * p<0.05 compared to controls.

There was a significant increase (p<0.001) in islet number per mm^2^ of pancreatic tissue in pregnant and lactating rats when compared to controls (Figure 5A). In addition, islet number was significantly greater (p<0.001) in pregnant as compared to lactating rats (Figure 5A). In keeping with this, the percentage of pancreas composed of islets was similarly increased (p<0.001) in pregnant rats compared to both control and lactating rats (Figure 5B). These changes were visualised in Figure 5C–E which also confirms the absence of any morphological abnormalities in the pancreas of pregnant or lactating rats compared to controls. As suggested from Figure 5C–E, individual islet area was significantly enlarged (p<0.05) in pregnant rats compared to control and lactating rats (Figure 6A) and there was a tendency for increased percentage of larger islets (>0.01 mm^2^) in pregnant and lactating rats (Figure 6B). There was no significant difference in islet alpha-cell area between groups (Figure 6C). Interestingly, further evaluation alpha-cell morphology using antibodies highly specific for glucagon and GIP revealed significant immunochemical staining and co-expression of glucagon and GIP in the majority of alpha-cells of all three groups of rats (Figure 6D–E; representative images taken from lactating rats).

### Effects of pregnancy and lactation on intestinal weight and intestinal GIP

**Figure 7:**
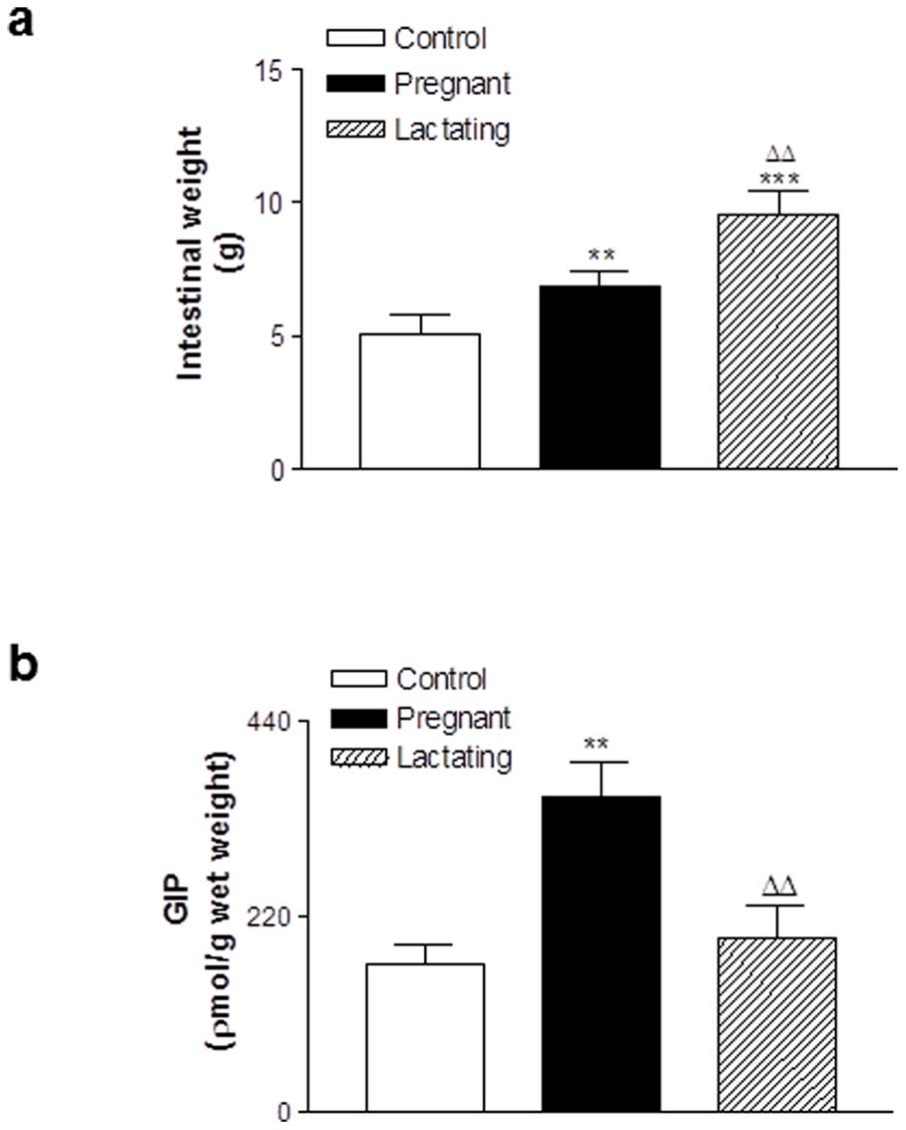
Effects of pregnancy and lactation on intestinal weight and GIP content in Wistar rats. Parameters were measured on days 21 and 42. Values are means ± SEM for 6 rats. ** p<0.01 and *** p<0.001 compared to controls. ^ΔΔ^ p<0.01 compared to pregnant rats.

Intestinal wet weight was significantly (p<0.01) increased in pregnant rats when compared to controls (Figure 7A). In addition, lactating rats had significantly (p<0.01) increased intestinal weight compared to pregnant rats (Figure 7A). However, intestinal GIP content was significantly (p<0.01) elevated in pregnant, but not lactating, rats when compared to controls (Figure 7B).

### Effects of pregnancy and lactation on abdominal adipose and mammary gene expression

The expression of ESS-RA and Prl-R were significantly (p<0.001) increased in abdominal adipose tissue of pregnant rats compared to controls, while lactation resulted in significantly (p<0.001) reduced expression of these genes when compared to pregnancy (Table 2). In addition, genes involved in lipolysis (HSL, HSD-1 and GCG-R) also exhibited significantly (p<0.001) increased expression in abdominal adipose tissue of pregnant rats, whereas lactation again resulted in a significant (p<0.001) decrease when compared to pregnant rats (Table 2). The expression of key genes involved in lipogenesis (LPL, Fat-P, GLUT4, ACC-1 and GIP-R) were not significantly altered in abdominal adipose tissue of pregnant rats (Table 2). However, lactation was associated with a significant (p<0.001) increase in expression of ACC-1 in abdominal adipose tissue when compared to both pregnant and control rats (Table 2). In mammary tissue, the expression of genes for ESS-RA, Prl-R, Fat-P, GLUT4, ACC-1, GIP-R, HSL, HSD-1 and GCG-R were significantly (p<0.01 to p<0.001) elevated in lactating rats when compared to both control and pregnant rats (Table 3). LPL was significantly (p<0.001) elevated in pregnant and lactating rats compared to controls (Table 3). In addition, pregnancy increased (p<0.001) the expression of ESS-RA, LPL, Fat-P, GLUT4, GIP-R, HSD-1 and GCG-R in mammary tissue of pregnant rats compared to controls (Table 3).

**Table 2:** Effects of pregnancy and lactation on gene expression in abdominal adipose tissue.

Gene	Control	Pregnancy	Lactation
**Hormone receptors**			
ESS-RA	1.0±0.4	10.9±0.2 ***	0.1±0.1 ***^,ΔΔΔ^
ESS-RB	1.0±0.5	1.8±0.1	1.6±1.1
Prl-R	1.0±1.4	100.4±1.0 ***	1.1±1.4 ^ΔΔΔ^
**Lipogenesis**			
LPL	1.0±0.4	2.5±0.6	0.1±1.0
Fat-P	1.0±0.4	0.2±0.3	0.1±1.0
GLUT4	1.0±0.7	0.1±0.1	0.1±0.1
ACC-1	1.0±0.8	5.9±0.2	448.4±10.3 ***^,ΔΔΔ^
GIP-R	1.0±1.0	1.7±0.2	0.1±0.1
**Lipolysis**			
HSL	1.0±0.8	456.7±4.9 ***	159.8±9.3 ***^,ΔΔΔ^
HSD-1	1.0±0.4	4.6±0.2***	0.1±0.1 ^ΔΔΔ^
GCG-R	1.0±0.8	24.7±1.8 ***	0.3±1.0 ^ΔΔΔ^

Values are means ± SEM for 4 rats. *** p<0.001 compared to controls. ^ΔΔΔ^ p<0.001 compared to pregnant rats.

**Table 3:** Effects of pregnancy and lactation on gene expression in mammary tissue.

Gene	Control	Pregnancy	Lactation
**Hormone receptors**			
ESS-RA	1.0±0.3	268.5±5.0 ***	2.63±1.2 ***^,ΔΔΔ^
ESS-RB	1.0±0.3	0.1±0.1	0.1±0.1
Prl-R	1.0±06	1.9±1.6	13.7±1.6 ***^,ΔΔΔ^
**Lipogenesis**			
LPL	1.0±0.4	121.7±4.5***	175.3±2.6 ***
Fat-P	1.0±1.4	36.7±4.0 ***	67.5±4.1 ***^,ΔΔΔ^
GLUT4	1.0±1.5	223.3±9.7 ***	18.4±2.5 ***^,ΔΔΔ^
ACC-1	1.0±0.6	1.8±2.5	13.7±1.6 ***^,ΔΔΔ^
GIP-R	1.0±0.3	4.8±0.5 ***	87.3±5.3 ***^,ΔΔΔ^
**Lipolysis**			
HSL	1.0±1.6	0.2±0.2	4.4±1.2 **^,ΔΔΔ^
HSD-1	1.0±1.2	154.5±4.46 ***	453.5±7.4 ***^,ΔΔΔ^
GCG-R	1.0±0.3	27.1±2.2 ***	247.5±8.0 ***^,ΔΔΔ^

Values are means ± SEM for 4 rats. ** p<0.01 and *** p<0.001 compared to controls. ^ΔΔΔ^ p<0.001 compared to pregnant rats.

### Body weight, circulating glucose and insulin and plasma and intestinal GIP in foetal and neonatal rats

**Figure 8:**
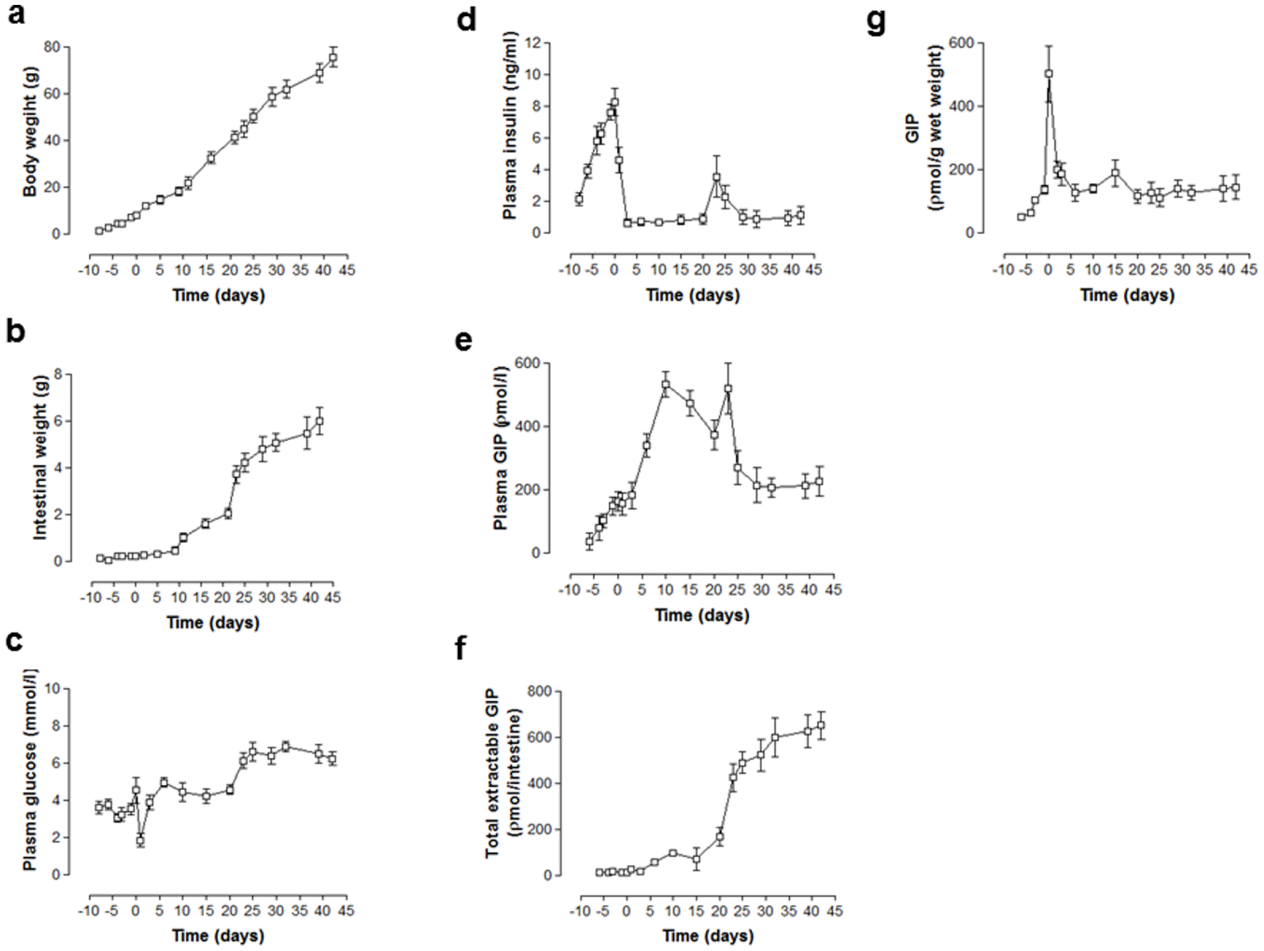
Effects of late foetal and neonatal life on (a) body and (b) intestinal weight, plasma (c) glucose, (d) insulin and (e) GIP, (f) total extractable intestinal GIP and (g) intestinal GIP content per g wet weight in Wistar rats. (**a–d**) Parameters were measured at various time points on days 10–21 of intrauterine life, 21 days during suckling and 21 days following weaning. (**e–g**) Parameters were measured at various time points on days 17–21 of intrauterine life, 21 days during suckling and 21 days following weaning. The day of birth is indicated by time zero. Values are means ± SEM for 5–6 rats.

As shown in Figure 8A&B, body and intestinal weights increased progressively over the study period in foetal and neonatal rats. Similarly, glucose levels increased during foetal life, during suckling at the progression to weaning at 21 days (Figure 8C). Notably, there was a rapid fall of plasma glucose at birth due to cessation of placental nutrition plus marked elevation of circulating insulin in the days preceding birth (Figure 8D). Insulin levels remained low during suckling but increased on introduction of solid food at 21 days. GIP was detectable in plasma of foetuses at 17 days gestation and rose steadily prior to birth (Figure 8E). Suckling was associated with markedly raised GIP concentrations, which declined to values similar to adult rats following weaning (Figure 8E). Total extractable intestinal GIP increased steadily, broadly in line with intestinal weight (Figure 8F). However, intestinal GIP content expressed as ρmol/g wet weight revealed a marked increase in GIP content in the period before birth followed by a sharp decline during lactation, when circulating GIP levels were high (Figure 8G).

## Discussion

Despite hyperphagia, pregnant rats exhibited lowered non-fasting glucose levels due to substantially raised plasma insulin concentrations that were independent of changes in circulating GIP. Similarly, glucose-stimulation of insulin, but not GIP, secretion was elevated in pregnancy, helping to maintain normal glucose tolerance despite coexistent insulin resistance [16]. The enhanced prolactin signalling in adipocytes, highlighted through increased prolactin receptor expression during pregnancy in the current study, may contribute to the development of insulin resistance [27]. Consistent with enhanced beta-cell functional demand, numbers and size of islets were increased in pregnant, compared to control and lactating, rats. Factors responsible for this are poorly understood but have been suggested to include prolactin, placental lactogens, progesterone and oestrogen [19]. Interestingly, it has recently been shown that proglucagon-derived peptides, such as glucagon-like peptide-1 (GLP-1), are not required for pregnancy-associated beta-cell proliferation [28]. Thus, the present evidence that most alpha-cells co-expressed GIP as well as glucagon is potentially interesting given well established effects of GIP on beta-cell proliferation and survival [20], [21]. Other studies have also demonstrated islet expression of GIP and provided firm evidence that GIP is synthesised and secreted by alpha-cells [29]. However, the role on intra-islet GIP is unclear and the present results did not suggest any difference in the expression of GIP or involvement in the altered islet morphology of pregnant, lactating or control rats.

**Figure 9:**
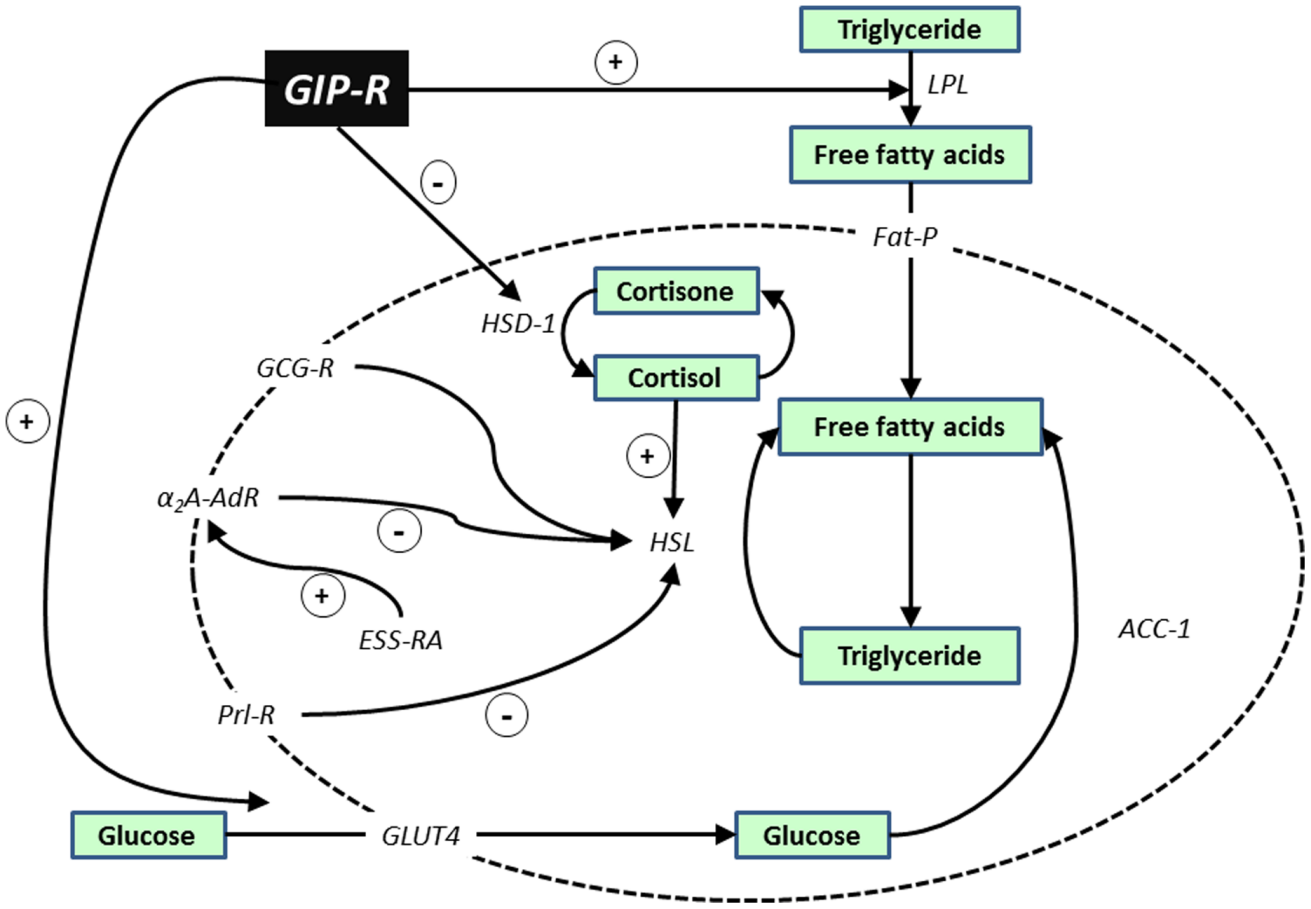
Simplified schematic depicting possible role of GIP-R and other key regulators in mammary and adipose tissue metabolism. It is envisaged that GIP increases uptake of free fatty acids and glucose by stimulating LPL and GLUT4 expression, favouring triglyceride formation. There is evidence that GIP also inhibits HSD-1 which, by limiting local cortisol production, restrains HSL and lipolysis. Other key actions on lipolysis via HSL are mediated by GCG-R, ESS-RA, Prl-R and adrenergic receptors [17], [30], [31]. α_2_A-AdR, α_2_ adrenergic receptor; acetyl CoA carboxylase-1, ACC-1; fatty-acid transport protein, Fat-P; glucagon receptor, GCG-R; GIP receptor, GIP-R; glucose transporter type 4, GLUT4; 11β-hydroxysteroid dehydrogenase type 1, HSD-1; hormone-sensitive lipase, HSL; lipoprotein lipase, LPL; oestrogen receptor A, ESS-RA; oestrogen receptor B, ESS-RB and prolactin receptor, Prl-R.

GIP is known to target adipose tissue and stimulate lipoprotein lipase, lipogenesis, fatty acid and glucose uptake, insulin-induced fatty acid incorporation whilst inhibiting glucagon and adrenergic receptor mediated lipolysis [9], [17], [30]. GIP also inhibits adipose tissue HSD-1 activity, which by decreasing local production of cortisol, also suppresses lipolysis induced by HSL activation [31]. These and other studies on the cellular mechanisms of adipocyte GIP action [8], [9], point to an important role of GIP in energy partition during lactation, presumably by increasing the availability of nutrients for milk production. Indeed, circulating GIP concentrations were substantially elevated in lactating, compared with control or pregnant, rats. This was associated with dramatic elevations in the expression of mammary tissue genes involved in lipogenesis and lipolysis during lactation, including GIP-R, Fat-P, GLUT4, ACC-1, HSL, HSD-1 and GCG-R. ESS-RA and Prl-R gene expression were also enhanced which is notable since activation of these receptors by circulating or locally produced oestrogen or prolactin has been shown to inhibit lipolysis in adipose tissue [27]. This would imply that basal circulating GIP, in concert with various other factors, has important actions on energy turnover in mammary tissue ensuring effective lipid production for milk during lactation, as depicted in Figure 9. Notably, in pregnancy the expression of genes for enzymes involved in the rate limiting-steps of lipogenesis and lipolysis, namely ACC-1 and HSL [32], [33], were unchanged or decreased in mammary tissue. Thus, the observed elevation in the expression of other lipogenic and lipolytic genes in this tissue during pregnancy presumably reflects adaptive responses during the gestational phase for pending lactation. In contrast, expression levels of ACC-1 and HSL was considerably increased in abdominal adipose tissue during lactation, with concomitant reductions in the level of expression of the other lipogenic and lipolytic genes studied. We presume that this is another adaptive response in abdominal adipose tissue, in order to reduce energy turnover and maintain the high energy needs of mammary tissue for milk production.

Despite the prominent hyperphagia and increased circulating GIP levels induced by lactation, intestinal GIP stores were not significantly elevated on day 21 of lactation. This probably reflects rapid turnover of cellular GIP from active K-cells, although other metabolic adaptations cannot be ruled out as lactation terminates [14]. Ironically, circulating GIP levels were normal in hyperphagic pregnant rats but intestinal concentrations were markedly elevated. Thus, pregnancy appears to be associated with tonic inhibition of GIP secretion leading to enhanced intestinal GIP storage, whereas lactation evokes markedly enhanced secretion of GIP. These processes and their underlying mechanisms could signify important metabolic adaptations during pregnancy and in the transition to lactation.

Reports suggest that the increase of circulating GIP concentrations as lactation progresses may not be entirely due to increased food intake *per se*, but also reflects endocrine and metabolic adaptations associated with lactogenesis [34]. Consistent with this view circulating GIP was elevated during lactation and then fell rapidly when milk secretion was terminated [14]. Our data in pregnant and lactating rats clearly show dissociation between hyperphagia and GIP concentrations. Thus, elevated concentrations of GIP seem indicative of a specific role in lactation, independent of normal anabolic and insulinotropic effects of GIP. Furthermore, in the current study plasma insulin concentrations correlated positively with plasma glucose levels, but not with GIP, suggesting an important extrapancreatic effect of GIP during lactation. As suggested above, it is likely that GIP has a function related to partition of nutrients for milk production. Thus, the largely gluco-regulatory hormone GIP [9], appears to have a key role in determining energy provision during lactation.

Observations in the offspring of pregnant rats also support an important role of GIP in foetal and neonatal development. Thus, GIP was measurable in intestines and plasma of foetuses from 17 days of gestation, broadly similar to detection of GIP mRNA and tissue GIP at 20 days [35], [36]. However, most notable was the marked and sustained elevation of plasma GIP in neonates from birth to weaning at 21 days. This corresponds to the period of suckling when fat content of milk can be expected to powerfully stimulate GIP secretion which will in turn provide deposition of triglyceride stores in adipose tissue. Interestingly, insulin concentrations remained low during suckling, but two prominent peaks were observed immediately prior to birth and following consumption of carbohydrate-rich diet on weaning. The former phenomenon is intriguing, being ascribed to transient release of a modified form of insulin from foetal pancreas [37]. However, another more recent explanation for increased insulin concentrations immediately prior to birth could be related to elevated levels of proinsulin and 32–33 split proinsulin [38]. This is dissociated from any surge of circulating GIP but may, together with interruption of placental nutrition, contribute to the prominent dip of plasma glucose observed around the time of birth [39].

In conclusion, this study has shown that pregnancy and the transition to lactation are associated with important metabolic adaptations including significant alterations in circulating GIP concentrations and both mammary and adipose tissue gene expression. Further studies are needed to delineate the extent to which the observed effects are due specifically to alterations in GIP receptor mediated effects. This might involve administration of neutralising antibodies or GIP antagonist, but this type of intervention in pregnant/lactating rats is difficult due to behavioural modification and the likely abortion or loss of litters. In addition, further investigation of the role of GIP in pregnancy and lactation could be performed in rodents with genetic knockout of the GIP receptor. Nonetheless, these data suggest a prominent role of intestinal K-cells and GIP in metabolic adaptation in mammary tissue and the partition of energy regulation during pregnancy, and particularly in transition to lactation.

## References

[1] Irwin N, Gault V, Flatt PR (2010) Therapeutic potential of the original incretin hormone glucose-dependent insulinotropic polypeptide: diabetes, obesity, osteoporosis and Alzheimer's disease? Expert Opin Investig Drugs 19: 1039–48. 10.1517/13543784.2010.513381 20698813

[2] Vella A, Rizza RA (2004) Extrapancreatic effects of GIP and GLP-1. Horm Metab Res 36: 830–6. 15655715 10.1055/s-2004-82617

[3] Usdin TB, Mezey E, Button DC, Brownstein MJ, Bonner TI (1993) Gastric inhibitory polypeptide receptor, a member of the secretin-vasoactive intestinal peptide receptor family, is widely distributed in peripheral organs and the brain. Endocrinology 133: 2861–70. 8243312 10.1210/endo.133.6.8243312

[4] Miyawaki K, Yamada Y, Ban N, Ihara Y, Tsukiyama K, et al (2002) Inhibition of gastric inhibitory polypeptide signaling prevents obesity. Nat Med 8: 738–42. 12068290 10.1038/nm727

[5] McClean PL, Irwin N, Cassidy RS, Holst JJ, Gault VA, et al (2007) GIP receptor antagonism reverses obesity, insulin resistance, and associated metabolic disturbances induced in mice by prolonged consumption of high-fat diet. Am J Physiol Endocrinol Metab 293: E1746–55. 17848629 10.1152/ajpendo.00460.2007

[6] Hansotia T, Maida A, Flock G, Yamada Y, Tsukiyama K, et al (2007) Extrapancreatic incretin receptors modulate glucose homeostasis, body weight, and energy expenditure. J Clin Invest 117: 143–52. 17187081 10.1172/JCI25483 PMC1705821

[7] Gaudin-Audrain C, Irwin N, Mansur S, Flatt PR, Thorens B, et al (2013) Glucose-dependent insulinotropic polypeptide receptor deficiency leads to modifications of trabecular bone volume and quality in mice. Bone 53: 221–30. 23220186 10.1016/j.bone.2012.11.039

[8] Irwin N, Flatt PR (2009) Evidence for beneficial effects of compromised gastric inhibitory polypeptide action in obesity-related diabetes and possible therapeutic implications. Diabetologia 52: 1724–31. 19533083 10.1007/s00125-009-1422-8

[9] Irwin N, Flatt PR (2013) *GIP* In: Handbook of Biologically Active Peptides. Kastin AJ: Eds. Elsevier: 2013: p. 1227–1236.

[10] Creutzfeldt W (2001) The entero-insular axis in type 2 diabetes – incretins as therapeutic agents. Exp Clin Endocrinol Diabetes 109: S288–303. 11460578 10.1055/s-2001-18589

[11] Durnin JV (1987) Energy requirements of pregnancy: an integration of the longitudinal data from the five-country study. Lancet 2: 1131–3. 2890029 10.1016/s0140-6736(87)91556-x

[12] Père MC, Etienne M (2007) Insulin sensitivity during pregnancy, lactation, and postweaning in primiparous gilts. J Anim Sci 85: 101–10. 17179545 10.2527/jas.2006-130

[13] Marks V (1985) How our food affects our hormones. Clin Biochem 18: 149–53. 3888442 10.1016/s0009-9120(85)80099-0

[14] Faulkner A, Martin PA (1998) Changes in the concentrations of glucagon-like peptide-1(7–36)amide and gastric inhibitory polypeptide during the lactation cycle in goats. J Dairy Res 65: 33–41. 10.1017/s0022029998002957 9718496

[15] Retnakaran R, Qi Y, Connelly PW, Sermer M, Hanley AJ, et al (2010) Low adiponectin concentration during pregnancy predicts postpartum insulin resistance, beta cell dysfunction and fasting glycaemia. Diabetologia 53: 268–76. 19937225 10.1007/s00125-009-1600-8 PMC2878328

[16] Bell AW, Bauman DE (1997) Adaptations of glucose metabolism during pregnancy and lactation. J Mammary Gland Biol Neoplasia 2: 265–78. 10882310 10.1023/a:1026336505343

[17] McIntosh CH, Widenmaier S, Kim SJ (2009) Glucose-dependent insulinotropic polypeptide (Gastric Inhibitory Polypeptide; GIP). Vitam Horm 80: 409–71. 19251046 10.1016/S0083-6729(08)00615-8

[18] Baggio LL, Drucker DJ (2007) Biology of incretins: GLP-1 and GIP. Gastroenterology 132: 2131–57. 17498508 10.1053/j.gastro.2007.03.054

[19] Sorenson RL, Brelje TC (1997) Adaptation of islets of Langerhans to pregnancy: beta-cell growth, enhanced insulin secretion and the role of lactogenic hormones. Horm Metab Res 29: 301–7. 9230352 10.1055/s-2007-979040

[20] Trümper A, Trümper K, Trusheim H, Arnold R, Göke B, et al (2001) Glucose-dependent insulinotropic polypeptide is a growth factor for beta (INS-1) cells by pleiotropic signaling. Mol Endocrinol 15: 1559–70. 11518806 10.1210/mend.15.9.0688

[21] Trümper A, Trümper K, Hörsch D (2002) Mechanisms of mitogenic and anti-apoptotic signaling by glucose-dependent insulinotropic polypeptide in beta(INS-1)-cells. J Endocrinol 174: 233–46. 12176662 10.1677/joe.0.1740233

[22] Irwin N, Montgomery IA, Moffett RC, Flatt PR (2013) Chemical cholecystokinin receptor activation protects against obesity-diabetes in high fat fed mice and has sustainable beneficial effects in genetic ob/ob mice. Biochem Pharmacol 85: 81–91. 23085436 10.1016/j.bcp.2012.10.008

[23] Abramoff MD, Magelhaes PJ, Ram SJ (2004) Image processing with image J. Biophotonics Int. 11: 36–42.

[24] Flatt PR, Bailey CJ (1981) Abnormal plasma glucose and insulin responses in heterozygous lean (ob/+) mice. Diabetologia 20: 573–7. 7026332 10.1007/BF00252768

[25] Morgan LM, Morris BA, Marks V (1978) Radioimmunoassay of gastric inhibitory polypeptide. Ann Clin Biochem 115: 172–7. 10.1177/000456327801500138 677795

[26] Irwin N, Francis JM, Flatt PR (2011) Alterations of glucose-dependent insulinotropic polypeptide (GIP) during cold acclimation. Regul Pept 167: 91–6. 21146561 10.1016/j.regpep.2010.12.001

[27] Brandebourg T, Hugo E, Ben-Jonathan N (2007) Adipocyte prolactin: regulation of release and putative functions. Diabetes Obes Metab 9: 464–76. 17587388 10.1111/j.1463-1326.2006.00671.x

[28] Sugiyama C, Yamamoto M, Kotani T, Kikkawa F, Murata Y, et al (2012) Fertility and pregnancy-associated ß-cell proliferation in mice deficient in proglucagon-derived peptides. PLoS One 7: e43745. 22928026 10.1371/journal.pone.0043745 PMC3426535

[29] Fujita Y, Wideman RD, Asadi A, Yang GK, Baker R, et al (2010) Glucose-dependent insulinotropic polypeptide is expressed in pancreatic islet alpha-cells and promotes insulin secretion. Gastroenterology 138: 1966–75. 20138041 10.1053/j.gastro.2010.01.049

[30] Mohammad S, Ramos LS, Buck J, Levin LR, Rubino F, et al (2011) Gastric inhibitory peptide controls adipose insulin sensitivity via activation of cAMP-response element-binding protein and p110ß isoform of phosphatidylinositol 3-kinase. J Biol Chem 286: 43062–70. 22027830 10.1074/jbc.M111.289009 PMC3234864

[31] Gögebakan Ö, Andres J, Biedasek K, Mai K, Kühnen P, et al (2012) Glucose-dependent insulinotropic polypeptide reduces fat-specific expression and activity of 11ß-hydroxysteroid dehydrogenase type 1 and inhibits release of free fatty acids. Diabetes 61: 292–300. 22179810 10.2337/db10-0902 PMC3266397

[32] Kraemer FB, Shen WJ (2006) Hormone-sensitive lipase knockouts. Nutr Metab (Lond) 3: 12. 16472389 10.1186/1743-7075-3-12 PMC1391915

[33] Lenhard JM (2011) Lipogenic enzymes as therapeutic targets for obesity and diabetes. Curr Pharm Des 17: 325–31. 21375498 10.2174/138161211795164185

[34] Relling AE (2007) Reynolds (2007) CK. Plasma concentrations of gut peptides in dairy cattle increase after calving. J Dairy Sci 90: 325–30. 17183100 10.3168/jds.S0022-0302(07)72633-4

[35] Gespach C, Bataille D, Jarrousse C, Rosselin G (1979) Ontogeny and distribution of immunoreactive gastric inhibitory polypeptide (IR-GIP) in rat small intestine. Acta Endocrinol (Copenh) 90: 307–16. 419918 10.1530/acta.0.0900307

[36] Higashimoto Y, Liddle RA (1994) Developmental expression of the glucose-dependent insulinotropic polypeptide gene in rat intestine. Biochem Biophys Res Commun 201: 964–72. 8003038 10.1006/bbrc.1994.1796

[37] Watts C, Gain KR (1984) Insulin in the rat fetus. A new form of circulating insulin. Diabetes 33: 50–6. 6360767 10.2337/diab.33.1.50

[38] Lindsay RS, Walker JD, Halsall I, Hales CN, Calder AA, et al (2003) Insulin and insulin propeptides at birth in offspring of diabetic mothers. J Clin Endocrinol Metab. 88: 1664–71. 10.1210/jc.2002-021018 12679454

[39] Snell K, Walker DG (1978) Glucose metabolism in the newborn rat: the role of insulin. Diabetologia 14: 59–64. 627334 10.1007/BF00429709

